# Anterior Cruciate Ligament Rupture with Concurrent Gastrocnemius Tear: A Case Report of a Rare Pattern of Injury †

**DOI:** 10.3390/reports9010022

**Published:** 2026-01-09

**Authors:** Su Jin Lee, Khang Duy Ricky Le, Roger Davies

**Affiliations:** 1 Department of Radiology, Adelaide MRI, Adelaide, SA 5031, Australia; 2 Department of General Surgical Specialities, The Royal Melbourne Hospital, Melbourne, VIC 3052, Australia; 3 Department of Medical Education, Melbourne Medical School, The University of Melbourne, Melbourne, VIC 3000, Australia; 4 School of Medicine, Faculty of Health, Deakin University, Geelong, VIC 3220, Australia

**Keywords:** anterior cruciate ligament injury, gastrocnemius tear, sporting injury, orthopaedic surgery, sprots medicine

## Abstract

**Background, Clinical Significance:** Anterior cruciate ligament (ACL) injuries are common, however the occurrence of concurrent gastrocnemius muscle tears is exceptionally rare. Given this, the diagnosis and management of this pattern of injury is poorly characterised, with lack of current clinically relevant classification systems and evidence-based guidelines to guide treatment. Early recognition is essential. with advanced imaging critical to guiding the diagnosis and management of patients with this pattern of injury. **Case presentation:** A 39 year old man presented with acute right knee swelling, pain and difficulty weightbearing following a sports-related fall. Clinical examination was suspicious for an ACL injury. Magnetic Resonance Imaging (MRI) of the knee demonstrated the disrupted and displaced ACL fibres, with extensive peri-cruciate oedema around the expected position of the ACL. It was associated with partial avulsion of the medial gastrocnemius origin and incomplete avulsion of the lateral gastrocnemius origin. The patient was referred for an urgent orthopaedics review and is currently on trial of conservative management. **Conclusions:** In this case report and review of the literature, we evaluate the current understanding of the complexities of combined musculoskeletal injuries and limitations of existing classifications in providing accurate diagnosis and management strategies. Given the rarity of this presentation, the case underscores the lack of evidence-based recommendations for early management, particularly in young, active individuals who are at risk of significant long-term functional impact.

## 1. Introduction and Clinical Significance

Anterior cruciate ligament (ACL) injuries are common sports related injuries to the knee. Despite this, the combination of ACL injury with concurrent gastrocnemius muscle tear is exceptionally rare and therefore the best-practice approaches for diagnosis and management remain highly heterogeneous. This case report and review of the literature highlights a case of this rare pattern of injury and through a literature review of the current evidence base, explores the complexities of biomechanical principles, diagnostic approaches and management for this injury. 

## 2. Case Presentation

A 39 year old man was referred by his general practitioner with right knee pain associated with a reduced range of movement following a fall whilst playing cricket. He is otherwise healthy with an unremarkable medical history, including no prior injuries to the right knee. He reported that he landed on the lateral aspect of the knee when it was internally rotated. He denies recall about any hyperextension-type mechanism of injury. On clinical examination, the patient was not able to weight-bear on the right knee. There was a prominent swelling of the right knee joint with tenderness on palpation. Range of motion was significantly restricted, particularly on extension. The Lachman test was positive, raising a strong suspicion of possible ligamentous or meniscus injury.

**Figure 1:**
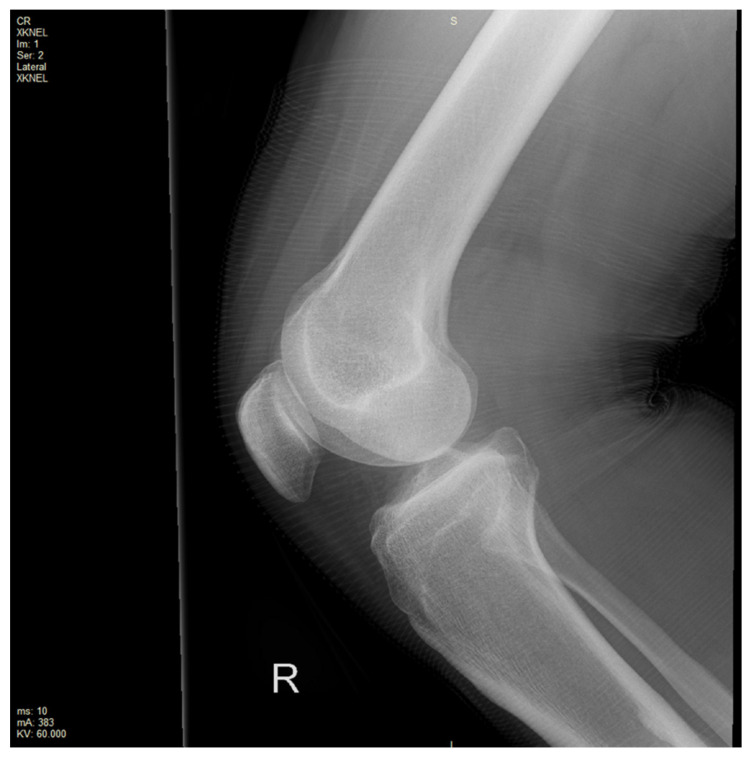
X-ray of the right knee with a soft tissue shadow extending into the supra-patellar recess.

**Figure 2:**
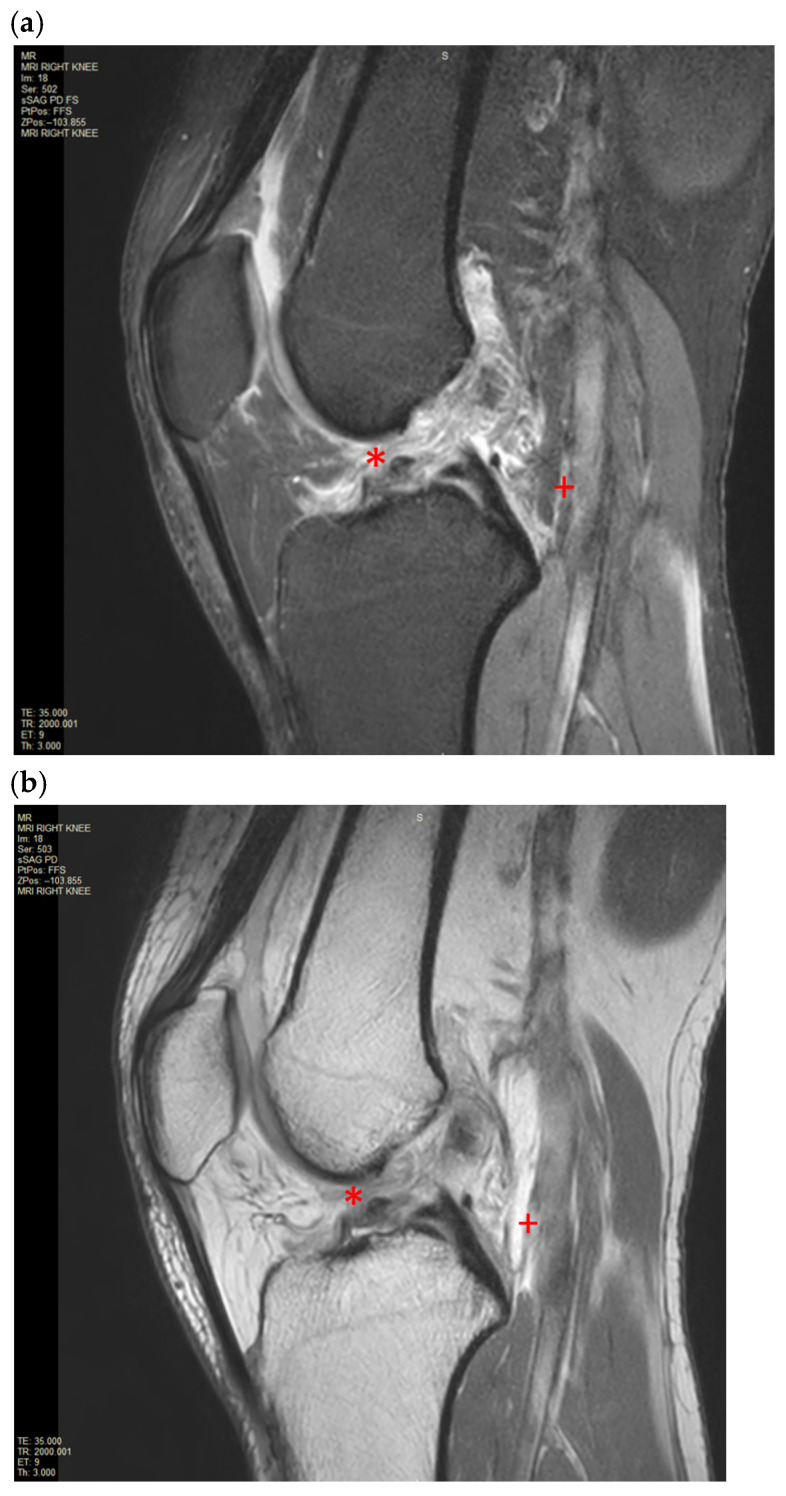
(**a**,**b**). Magnetic resonance imaging (MRI) of the right knee image of the near complete disruption of anterior cruciate fibres (*) associated with evidence of recent medial and lateral gastrocnemius origin incomplete disruption (+) on Sagittal PD SPAIR and PD TSE.

**Figure 3:**
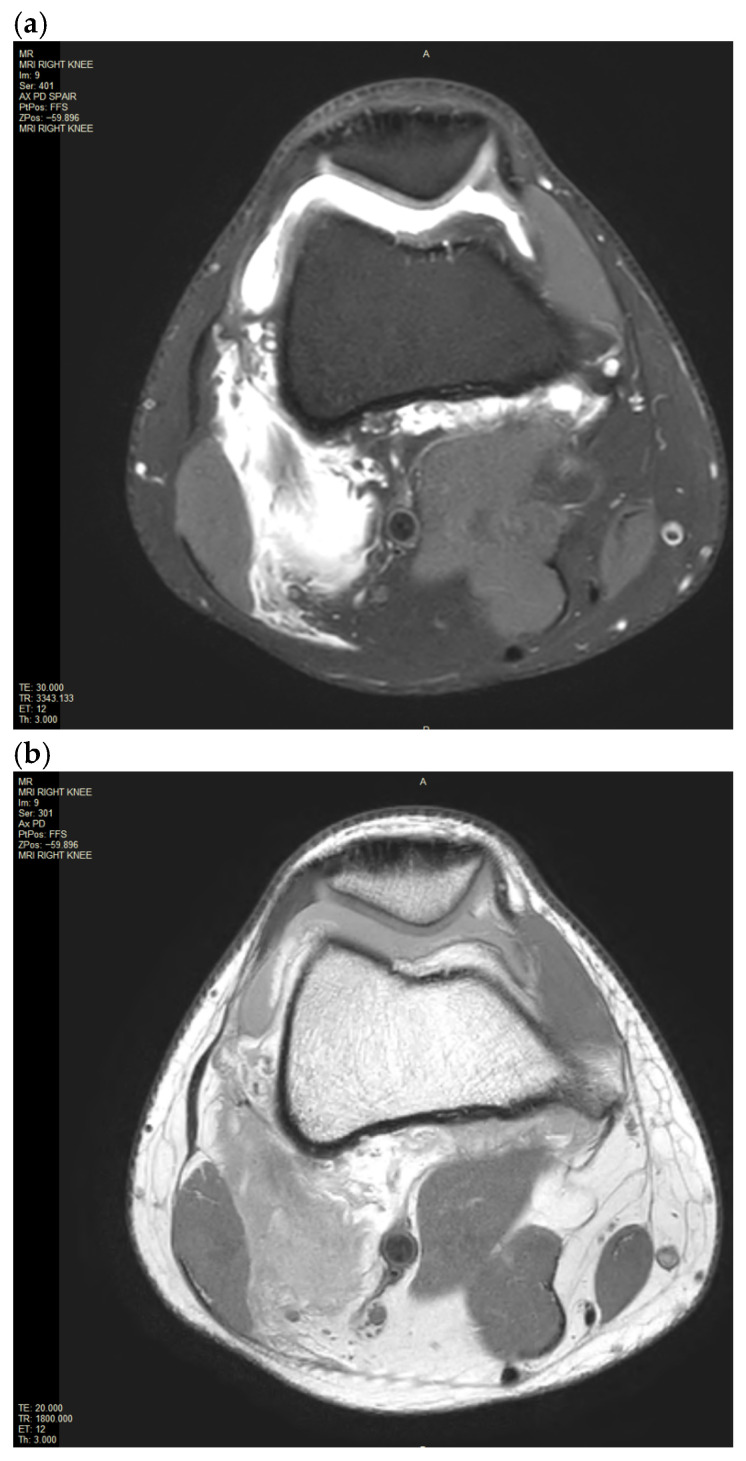
(**a**,**b**). MRI of the right knee image of the moderate knee joint effusion into the supra-patellar recess associated with incomplete avulsion of the lateral gastrocnemius origin as well as intramuscular haemataoma and partial avulsion of the medial gastrocnemius origin on Coronal PD PD SPAIR and PD TSE.

An X-ray of the right knee was initially performed to rule out a bony injury (Figure 1). The X-ray demonstrated marked soft tissue swelling of the knee joint with moderate effusion extending into the supra-patella recess. There was no evidence of acute or recent bony injury. The presence of radiological joint effusion raised suspicion for potential ligamentous or meniscal injuries. Subsequently, a magnetic resonance image (MRI) of the right knee was performed to further characterise the injury. MRI was obtained with a 1.5T Philips Achieva dStream MRI (Philips, Amsterdam, Netherlands) with Release 5.7 software. The routine knee protocol was performed, including sequences as follows: Axial Proton Density T2-weighted Turbo Spin Echo (Axial PD TSE), Proton Density Spectral Attenuated Inversion Recovery (PD SPAIR), Sagittal PD TSE, PD SPAIR, Coronal PD TSE, PD SPAIR (Figure 2a,b and Figure 3a,b).

The MRI of the right knee confirmed a moderate joint effusion extending into the supra-patella recess on the right with a focal intra-articular fluid located anterior to the tibial insertion of the ACL and posterior to the posterior cruciate ligament (PCL). The disrupted ACL fibres were displaced and concertinaed inferiorly. There was extensive pericruciate oedema around the expected position of the ACL. There was a focal bone oedema in relation to the central sulcus of the lateral femoral condyle. There was also partial avulsion of the medial gastrocnemius origin and incomplete avulsion of the lateral gastrocnemius origin. There was an extensive high fluid signal in the medial and lateral gastrocnemius origin at the posterior medial and lateral femoral condyle with an associated longitudinal tear. There were no injuries to the primary posterolateral corner structures, such as the lateral collateral ligaments or the popliteus tendon.

The patient was reviewed by the orthopaedic surgeon and is currently on trial of conservative management.

## 3. Discussion

The ACL is the most injured ligament in the knee. The overall incidence of the ACL injury is unclear in the Australian population; however, the incidence in the United States alone is approximately 1 in 3500 people [1]. ACL injury is more common in women and primarily occurs by non-contact mechanisms. A direct impact to the lateral aspect of the knee, as shown in this case presentation, has also been encountered as an injury mechanism [1]. ACL injury is often associated with meniscal injury and medial collateral ligament injury, known as the “Unhappy triad” [2].

Although ACL injury can be diagnosed clinically, MRI is the primary diagnostic modality with a sensitivity of 86% and a specificity of 95% [1]. In addition, MRI has co-benefits of characterising local anatomy and confirming additional local injuries to guide management strategies. Invasive procedures, including knee arthroscopy, can also be used to further characterise the severity of tears, which are seldom utilised as the primary modality of diagnosis due to their invasive nature. Furthermore, arthrography is considered the gold standard; however, it is similarly limited as an initial investigation due to its accessibility, cost and anaesthetic risks [1]. In such cases where anaesthetic risk outweighs the benefit of proceeding, an MR arthrogram may also be considered the gold standard.

Central to the biomechanical mechanisms of this pattern of injury are the antagonistic effects of the gastrocnemius muscles with the ACL. As a biarticular muscle that crosses the knee joint, the contraction of the gastrocnemius muscle can generate posterior femoral translation relative to the tibia and, at the same time, particularly at times of extension or dynamic landing, increase anterior tibial shear force and therefore ACL strain [3,4]. Although theoretically and biomechanically, this antagonism may describe why the pattern of injury described in this case occurs, it remains exceedingly rare. In cases of injury, as for ours, the mechanisms by which this occurs can only be postulated. In particular, it is likely it has arisen as a result of excessive force and potential hyperextension, which subjects the gastrocnemius as well as the cruciate ligaments to excessive force and potential for injury [5]. Despite this, we were unable to ascertain in clinical history the exact biophysical mechanisms, indicating a potential for recall bias.

In our case, an MRI of the knee confirmed near-complete ACL disruption associated with the incomplete disruption of the medial and lateral gastrocnemius muscles. This combined injury is extremely rare, with no prior cases documented in the literature at the time of initial writing. However, a recent case by Li et al. (2025) reports on a similar pattern of injury [6]. The authors managed this patient successfully with a staged surgical approach with primary posterior capsular and gastrocnemius repair followed by secondary anterior and posterior cruciate ligament reconstruction [5]. Interestingly, the authors also comment on the rarity of this condition, with no prior cases noted in the literature. In part, this may be explained by other protective mechanisms, such as the role of the hamstring muscles and soleus in absorbing force from high-stress activities such as landing dynamically on a single leg [7]. Given this, there is currently no grading system that exists for this type of muscle injury and therefore a lack of clearly defined management pathways for clinicians in approaching gastrocnemius injuries in the presence of synchronous ACL rupture. Nonetheless, muscle injuries are common in sports [3]. Muscle injury classification systems exist and are designed to assist clinicians with decision making, particularly in stratifying patients for conservative or operative interventions. However, these classification systems have been criticised for their limited diagnostic accuracy and therefore prognostic significance to clinicians. Recently, the British Athletics Muscle Injury Classification system was developed as an alternative grading system which included radiological MRI features to provide a suitable diagnostic framework for grading muscular injuries to guide treatment [6]. The system outlines five types of muscle injuries (Grade 0–4) based on the anatomical site of muscle injury on MRI [6]. According to the classification, the medial and lateral gastrocnemius tears shown in this MRI would be assessed as grade 3 injuries. Nevertheless, the relevance of this grading for clinicians remains poorly validated, with a lack of real-world evidence translating these grading systems to clear treatment pathways and positive outcomes for patients. For this pattern of injury whereby there is concurrent medial and lateral gastrocnemius injury in the presence of ACL rupture, further research is required into informing relevant grading guidelines, incorporating radiological and clinical features to guide best practice management of these injuries.

## 4. Conclusions

This case presentation highlights the rarity of combined injuries, including ACL disruption and gastrocnemius muscle tears. Currently, no specific classification system is available to grade the severity of this type of injury pattern. Although MRI serves as the primary diagnostic modality for identifying ACL injuries and associated injuries, the current muscle injury classification systems are limited in diagnostic accuracy and therefore clinical relevance. While isolated gastrocnemius tears are typically managed conservatively, the presence of a concomitant ACL rupture introduces additional considerations. Patients with combined injuries may experience prolonged functional impairment due to instability and muscular dysfunction. In young, highly active individuals, early surgical reconstruction may be beneficial to restore stability and reduce the risk of secondary meniscal or chondral damage. Our case advocates further research into the development of a novel classification system that can effectively address the complexities of combined musculoskeletal injuries. This case adds to the understanding of such uncommon injury patterns and underscores the importance of accurate diagnosis and management strategies.

## Data Availability

The original contributions presented in this study are included in the article. Further inquiries can be directed to the corresponding author.
